# Supplementary material for: Combining antimiR-25 and cGAMP Nanocomplexes Enhances Immune Responses via M2 Macrophage Reprogramming
Source: Int J Mol Sci. 2024 Nov 28;25(23):12787. doi: 10.3390/ijms252312787 (PMC11641323; doi:10.3390/ijms252312787)
Supplement: Supplementary file 1 [file ijms-25-12787-s001.zip › ijms-3269200-supplementary.pdf]

Supplementary data

Table S1: qPCR primers

	Primer sequences	
Gene name	Forward sequence	Reverse sequence
Gapdh	TCCATGACAACTTTGGCATTG	CAGTCTTCTGGGTGGCAGTGA
Eef1a1	TCCACTTGGTCGCTTTGCT	CTTCTTGTCCACAGCTTTGATGA
Il1b	ACCCTGCAGCTGGAGAGTGT	CCATCTTCTTCTTTGGGTATTGCTT
Mrc1	TGTCAACCCTGCAGATTTCAAG	GAGTGGCTTACGTGGTTGTTTC
Arg1	GCAGAGGTCCAGAAGAATGG	AGCATCCACCCAAATGACAC
Ifna	CCTGAGAGAAGAAACACAGCC	TTCTGCTCTGACCACCTCCC
Il1b	ACCCTGCAGCTGGAGAGTGT	CCATCTTCTTCTTTGGGTATTGCTT
Cccl10	GTCTGAGTGGGACTCAAGGGATC	CAGTTGCAGCGGACCGTC

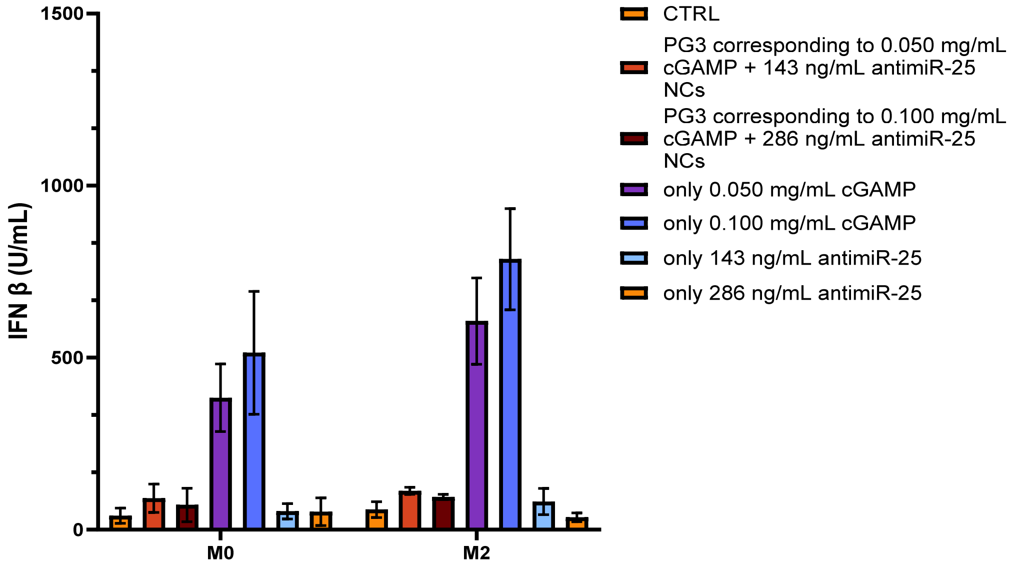

Figure S1. Characterization of SB28-derived EVs from hypoxic culture. Transmission Electron Microscopy (TEM) imaging of EVs secreted by SB28 GBM cells. Pictures are representative of at least 6 images. Electron cryo-microscopy (cryo-EM) imaging of EVs secreted by SB28 GBM cells. Pictures are representative of at least 3 images. Western blot analysis of cells and EVs from SB28 cell line.

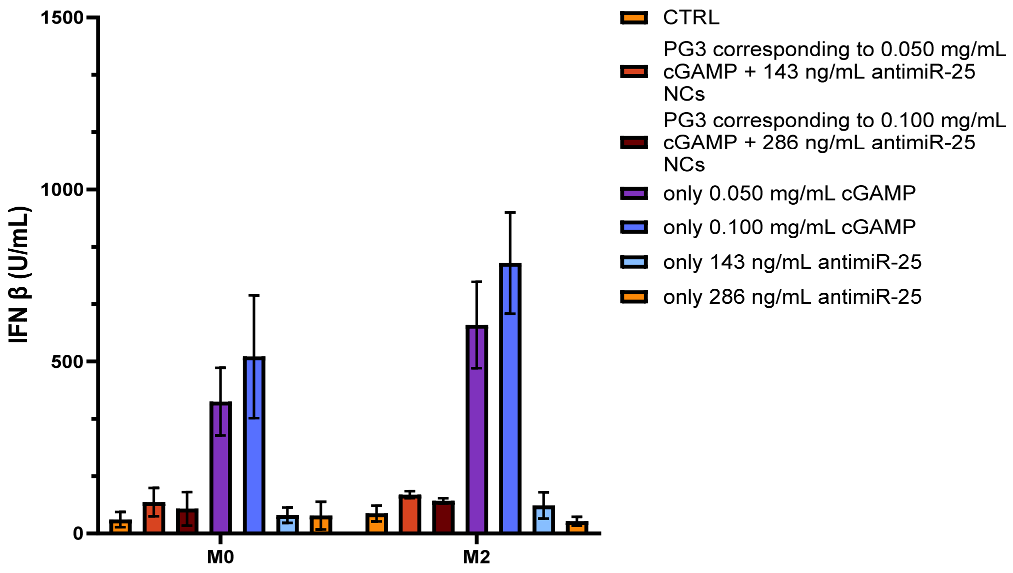

Figure S2. NTA size results of PG3 alone and complexed with antimiR-25 at two NP ratios 1:1 and 2:1, respectively.

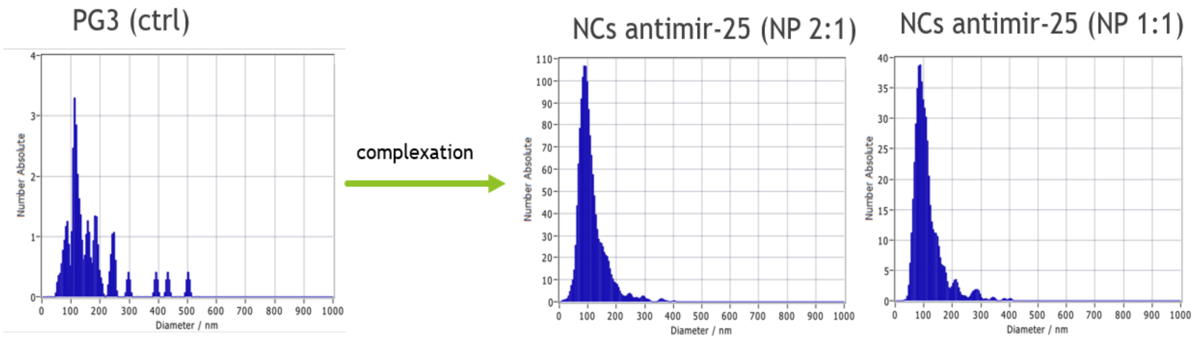

Figure S3. cGAMP alone induces IFN-β secretion in M0 and M2 macrophages. Levels of IFN-β secretion of BMDMs polarized to M0 (A) and M2 (B) upon incubation in physioxic (5% O2) were measured. The BMDMs were treated with vehicle (CTRL), antimiR-25 NCs (PG3) or antimiR-25 (NCs free or cGAMP (NCs free).

(A)

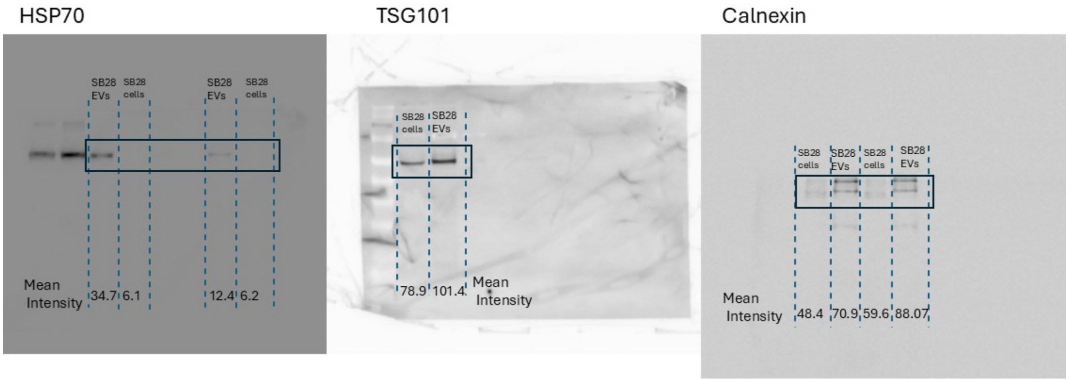

(B)

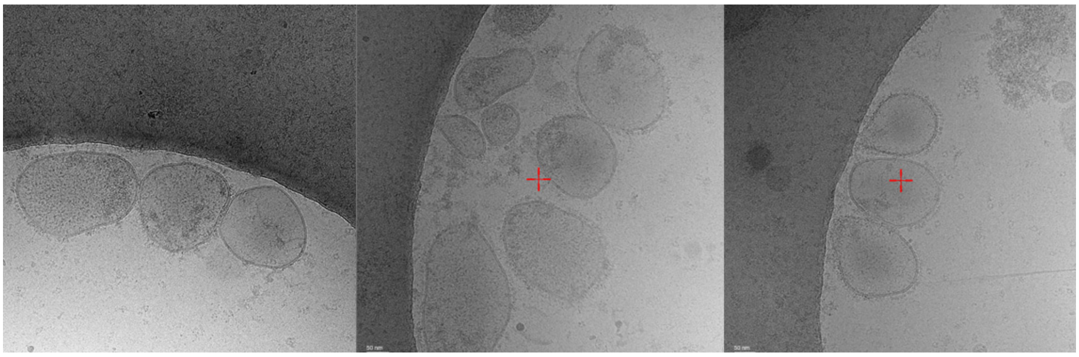

Figure S4. Additional Western blot and TEM imaging analysis. (A) Quantitative analysis of raw Western blot membranes performed using ImageJ software. (B) Transmission electron microscopy (TEM) analysis of extracellular vesicles (EVs) derived from SB28 cells, including size measurements, conducted using OLYMPUS software.
